# Use of Deferasirox Film-Coated Tablets in Pediatric Patients with Transfusion Dependent Thalassemia: A Single Center Experience

**DOI:** 10.3390/biology11020247

**Published:** 2022-02-05

**Authors:** Alkistis Adramerina, Nikoleta Printza, Emmanouel Hatzipantelis, Symeon Symeonidis, Labib Tarazi, Aikaterini Teli, Marina Economou

**Affiliations:** 1 1st Pediatric Department, School of Medicine, Faculty of Health Sciences, Aristotle University of Thessaloniki, 54642 Thessaloniki, Greece; nprintza@gmail.com (N.P.); ssymeoni@gmail.com (S.S.); teligaik@yahoo.gr (A.T.); kely@auth.gr (M.E.); 2 2nd Pediatric Department, School of Medicine, Faculty of Health Sciences, Aristotle University of Thessaloniki, 54621 Thessaloniki, Greece; chatzipa@med.auth.gr; 3 Tomografia AE, Medical Center, 54622 Thessaloniki, Greece; tomographia@tomographia.gr

**Keywords:** deferasirox, thalassemia, iron overload, chelation, pediatric patients

## Abstract

**Simple Summary:**

Thalassemia is a hereditary anemia characterized by defect in hemoglobin synthesis. Patients with severe forms of the disease require regular blood transfusions, as well as iron chelation. Available chelators have demonstrated long-term efficacy and safety, but present various limitations. A new deferasirox film-coated tablet (DFX FCT) was designed to offer a more convenient mode of administration and better tolerability. The present study aimed to assess the efficacy and safety of DFX FCT in thalassemic pediatric patients. DFX FCT proved safe for older patients, but demonstrated increased frequency of adverse events in younger patients, resulting in drug discontinuation. As for efficacy, DFX FCT succeeded in maintaining a stable iron load, though it required use of relatively high doses.

**Abstract:**

Thalassemic syndromes are characterized by clinical heterogenicity. For severe disease forms, lifelong blood transfusions remain the mainstay of therapy, while iron overload monitoring and adequate chelation treatment are required in order to ensure effective disease management. Compared to previous chelators, the new deferasirox film-coated tablet (DFX FCT) is considered to offer a more convenient and well-tolerated treatment scheme, aiming at better treatment-related and patient-related outcomes. The present study’s objective was to prospectively evaluate the safety and efficacy of DFX FCT in children and adolescents with transfusion-dependent thalassemia. Data collected included patient demographics, hematology and biochemistry laboratory work up, magnetic resonance imaging of heart and liver for iron load, as well as ophthalmological and audiological examination prior to and a year following drug administration. Study results confirmed DFX FCT safety in older children in a manner similar to adults, but demonstrated increased frequency of adverse events in younger patients, mainly, involving liver function. With regards to efficacy, study results confirmed the preventive role of DFX FCT in iron loading of liver and heart, however, higher doses than generally recommended were required in order to ensure adequate chelation.

## 1. Introduction

Thalassemia syndromes are recessively inherited hemoglobinopathies, characterized by defects in hemoglobin production [1,2]. Mutations within globin genes, located in chromosomes 16 and 11, result in reduced production of globin chains and, thus, hemoglobin A [3]. Clinical presentation is heterogenous, ranging from complete absence of symptoms to severe anemia, characterized by ineffective erythropoiesis with bone expansion and extramedullary hematopoiesis [1]. Although hemopoietic stem cell transplantation is an available curative treatment, the mainstay of therapy remains lifelong blood transfusions [1,4].

Regular transfusions, in addition to excessive gastrointestinal iron absorption, contribute to iron overload over time [5]. Subsequent parenchymal iron deposition, primarily in the heart, liver and endocrine glands, results in severe organ damage [6]. Thus, besides safe transfusions, iron overload monitoring and iron chelation treatment are necessary in order to ensure effective disease management [2]. Pediatric patients require a comparatively larger transfusion load than adults so as to achieve normal growth and development. Hence, adequate chelation during childhood, i.e., dose tailoring based on changing body weight, as well as treatment modifications guided by iron overload markers, is of great importance in order to achieve complication free survival-even, normal life expectancy [7].

Iron load is, routinely, assessed by serum ferritin-availability and low cost of the measurement compensating for disadvantages, such as values being dependent on concurrent inflammation and type of chelator [5,8]. In the last few decades, additional use of magnetic resonance imaging (MRI) has allowed for non-invasive, direct evaluation of iron stores in target organs [9].

Iron chelation therapy (ICT) is, traditionally, initiated around the age of 2, after the first 10–20 transfusions or when ferritin value exceeds 1000 ng/mL [5,10,11]. ICT aims at maintaining safe iron levels in the body, by forming a complex with iron and promoting its excretion, ideally, with minimum related toxicity [10,12,13]. Given that the benefits of chelation therapy are not immediately perceived, as organ damage develops gradually over time, and that all chelators have certain limitations, adherence is often suboptimal. Therefore, a convenient and well-tolerated chelation regimen is important in order to achieve both the desired treatment-related and patient-related outcomes [9,14].

To date, three iron chelators are licensed: deferoxamine (DFO), deferiprone (DFP) and deferasirox (DFX) [6]. DFO is subcutaneously administered, requiring a several hour infusion each day. It has been widely used for four decades despite its limitations (suboptimal potential for compliance due to mode of administration, as well as side effects, especially, in the context of lower iron load) [7,15]. DFP has the advantage of being given orally, but is indicated as second line treatment [16]. The newest oral chelator, DFX, is licensed as first line treatment for children over 6 years of age, and as first line treatment in USA—but second line treatment in Europe—for children of younger age (2–6 years old) [6]. Given that DFX is administered once-daily, it offers an advantage with regards to potential for compliance and patient satisfaction when compared to both the parenterally administered DFO and the thrice-daily orally administered DFP [11,17].

Currently, there are two DFX formulations available. The original formulation is a dispersible tablet (DT), designed to be consumed on an empty stomach in the form of a suspension after mixing with water or juice. However, the preparation is a lengthy process, and the final oral suspension is not palatable and often related to reduced gastrointestinal tolerability. In addition, bad taste and large volume of the suspension often lead to the full amount not being consumed, especially by young patients. The new film-coated tablet (FCT) DFX formulation was developed in an effort to overcome these issues and, because of the use of the same active ingredient, was approved based on the clinical trials run for the original DFX formulation [13]. FCT lacks excipients (lactose and sodium sulfate) responsible for gastrointestinal effects, and can be taken with or without a light meal, offering a more convenient mode of administration [14].

When DFX FCT was licensed in Greece, a prospective study was designed, aiming to assess safety and efficacy of the new DFX formulation in pediatric patients with transfusion-dependent thalassemia either chelation naive or previously receiving DFX DT or another chelator.

## 2. Materials and Methods

### 2.1. Study Setting

The present study is a prospective observational study on DFX FCT safety and efficacy, administered to pediatric patients followed at the Thalassemic Unit for Children and Adolescents of Northern Greece. Study enrollment period lasted for 12 months. Approval by the Ethics Committee was obtained prior to study initiation. Written informed consent signed by parents/caregivers of children participating in the study was required prior to enrollment.

### 2.2. Subject Eligibility

Eligible for study participation were pediatric patients aged ≥ 2 years with any transfusion-dependent thalassemic syndrome; patients were eligible whether previously treated with any iron chelator or previously chelation-naive. Key exclusion criteria were: Glomerular filtration rate (GFR) < 60 mL/min, serum creatinine (sCr) > 1.5 × upper limit of normal (ULN), alanine (ALT) and/or aspartate aminotransferase (AST) > 5 × ULN.

### 2.3. Measures and Data Collection

Data collected included patient characteristics with regards to age, height and body weight, as well as data on previous chelation therapy. A laboratory work up was performed before and 12 months after DFX FCT administration, including cardiac MRI T2*, MRI liver iron concentration (LIC), urinary tract ultrasound, pure tone audiometry (PTA) testing, as well as fundoscopy.

In addition, before DFX FCT administration baseline values of ferritin, sCr, ALT, AST, bilirubin, alkaline phosphatase (ALP), urine calcium-to-creatinine and protein-to-creatinine ratio were recorded. GFR was estimated using the CKD-EPI Creatinine Equation, as recommended by the National Kidney Foundation. Cutoff value of proteinuria or calcinuria was urine protein-to-creatinine or calcium-to-creatinine ratio > 0.2. During the first month of DFX FCT therapy, sCr was measured weekly, and ALT, AST, bilirubin and ALP every 2 weeks. After the first month of treatment, all the above-mentioned biochemical markers were evaluated monthly for an 11-month period. In cases of dose modification, biochemical laboratory work up was repeated as per first dose.

Adverse events during study period were recorded in accordance to the summary of DFX FCT characteristics. 

### 2.4. Statistical Analysis 

All statistical tests were performed using the SPSS Statistics software for Windows. Spearman correlation analysis was used to assess the univariate correlations between the on-study variables. Independent-sample t-test was conducted to compare means in patient groups. A two-tailored *p* value of <0.05 was considered statistically significant.

## 3. Results

In total, 25 pediatric patients were enrolled. Male:female ratio was 16:9. Median patient age was 13 years (range 2–18), with 3 distinct age groups (2–6, >6–10, >10–18 years) being represented by 3 (12%), 5 (20%) and 17 (68%) patients, respectively. Prior to DFX FCT administration, 14 (56%) patients were on the previous DFX formulation (DFX DT), 7 (28%) were on a different iron chelator and 4 (16%) were chelation-naive (Table 1). 

At study entry median ferritin value was 1143 ng/mL (range 345–2253) and at the end of study 1117 ng/mL (range 318–3256) (*p* = 0.510). Mean ferritin value was negatively correlated to patient age, weight and height at study entry (rs = −0.576, *p* = 0.003, rs = −0.582, *p* = 0.002, rs = −0.605, *p* = 0.001). In specific, patients over 10 years of age presented with significantly lower mean ferritin values (*p* = 0.023).

Prior to enrollment, patients on DFX DT received a median dose of 20.5 mg/kg/d and, as recommended, were started on a dose reduced by 30% when switching to DFX FCT. At study entry median DFX FCT dose was 14.3 mg/kg/d (range 10–22.5) and at study end significantly increased (18 mg/kg/d, range 7.5–22.5) (*p* = 0.011). No correlation was found between mean patient DFX FCT dose and mean ferritin value (rs = 0.210 *p* = 0.314).

With regards to renal function, at study entry median sCr value and estimated GFR were 0.65 mg/dL (range 0.4–0.9) and 93 mL/min (range 72–141), respectively. At the end of the study median sCr and GFR values were 0.66 mg/dL (range 0.45–0.99) and 92 mL/min (range 71–139), respectively (*p* = 0.165 and *p* = 0.188). Five (20%) patients presented with a sCr increase of >33% during follow up compared to baseline values, with consequent reduction in GFR. Dose adjustments resulted in sCr recovery in four cases, whereas in one patient DFX discontinuation was deemed necessary. No correlation was found between mean patient sCr and mean patient DFX FCT dose (rs = −0.74 *p* = 0.727). Similarly, no correlation was found between sCr change and patient age (rs = −0.262 *p* = 0.2014), although patients presenting with a sCr increase of >33% had higher weight at study entry (*p* = 0.047).

At the beginning of study nine (36%) patients presented with a known calcinuria. At study entry median spot urinary protein and calcium:creatinine ratio were 0.06 and 0.16, respectively, whereas at study end median spot urinary protein and calcium: creatinine ratio were 0.06 and 0.21, respectively (*p* = 0.547 and *p* = 0.1). Increase in spot urinary protein beyond ULN was reported in 12 (48%) patients, with one patient presenting a consistently abnormal value. A calcium: creatinine ratio increase beyond ULN was reported in 24 (96%) patients, out of which 15 (60%) showed a consistently abnormal value. Renal ultrasound was normal in all patients at study entry, whereas after 12 months of DFX FCT administration 2 (8%) patients showed imaging signs of nephrolithiasis. Mean calcium:creatinine ratio presented no correlation either with mean sCr or mean ferritin (rs = −0.141 *p* = 0.5, rs = −0.245 *p* = 0.238) 

As to liver function, median AST and ALT values before DFX FCT initiation were 22.5 U/L (range 12–49) and 14 U/L (6–31), respectively. At some point during the study, elevation of AST and ALT was reported in 12 (48%) and 11 (44%) patients, respectively. In 8 cases dose adjustment was required until normalization of values, while in the remaining 3 patients DFX FCT was discontinued due to consistently abnormal liver enzyme levels of > 3 × ULN. All patients presenting transaminasemia belonged in the 2–6-year group. Median AST and ALT values at study end were 22 U/L (14–111) and 15 U/L (7–127), respectively (*p* = 0.173 and *p* = 0.076). No correlation was found between either mean AST or ALT and patient age (rs = −0.422 *p* = 0.36 and rs = −0.222 *p* = 0.286), nor with mean ferritin (rs = 0.332 *p* = 0.105 and rs = 0.279 *p* = 0.176). Median bilirubin levels at study entry and study end were 1.95 mg/dL (range 0.61–10.8) and 1.79 mg/dL (range 0.91–14.81), respectively (*p* = 0.089), including a patient with a known history of Gilbert syndrome. Finally, median ALP values were 137 U/L (range 71–506) and 159 U/L (range 59–445) at beginning and end of study, respectively (*p* = 0.710).

**Figure 1:**
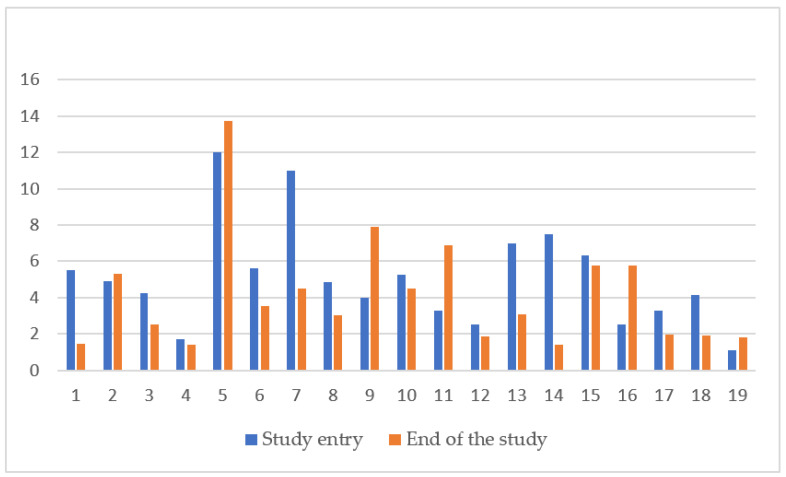
LIC change (mg/kg dry weight) in 19 patients prior and after 12 months of DFX FCT administration. LIC liver iron concentration.

**Figure 2:**
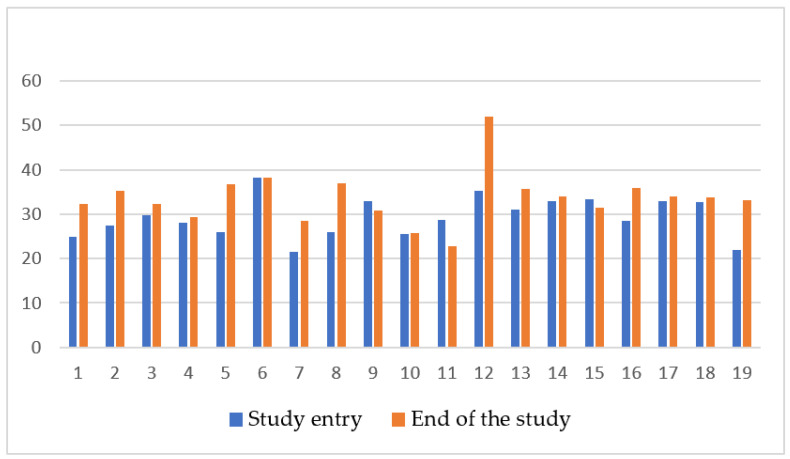
Cardiac T2* (ms) in 19 patients prior and after 12 months of DFX FCT administration.

Evaluation of liver and cardiac iron with MRI at the beginning and end of study was performed in 19 out of 25 (76%) patients, as younger patients requiring sedation for the procedure were excluded. At study entry median LIC was 4.86 mg/g dw (range 1.1–12) and at study end 4.01 mg/g dw (range 1.4–13.75) (*p* = 0.142). (Figure 1) Median cardiac MRI T2* at the beginning and end of study were 28.8 ms (range 21.5–38.3) and 33.5 ms (range 22.3–68.2), respectively (*p* = 0.006) (Figure 2). No correlation was found between LIC or cardiac T2* and mean ferritin (rs = −0.20 *p* = 0.935, rs = −0.225 *p* = 0.355) or mean DFX dose (rs = 0.155 *p* = 0.49 and rs = −0.429 *p* = 0.06). Laboratory measurements and imaging assessments are shown in Table 2.

PTA revealed a mild hearing loss in one (4%) patient at study end, while fundoscopy showed diffuse retinal pigmentation in another (4%) patient.

With regards to other drug-related adverse events, 5 (20%) cases were reported overall. In specific, 2 (8%) patients presented with diarrhea, one (4%) with nausea, one (4%) with vomiting and the remaining one (4%) with a skin rash. In all cases adverse events presented early in the course of DFX FCT administration and resolved quickly without intervention.

In total, 21/25 (84%) patients completed the 12-month DFX FCT therapy, whereas 4 (16%) patients were switched to another chelator because of adverse events. One (4%) of these patients was on a different chelator prior to study enrollment and 3 (12%) were chelation naive. No patient in the 2–6-year group completed the study.

## 4. Discussion

In thalassemic patients, the degradation of regularly transfused red blood cells by macrophages is followed by saturation of transferrin’s binding capacity by labile cellular iron, while excess iron circulates as non-transferrin bound iron (NTBI) [15]. NTBI is transported into hepatocytes, cardiac myocytes and endocrine glands, resulting in organ dysfunction [9,15,18]. Heart complications are the most important life-limiting complications attributed to iron overload and have been reported as cause of death in up to 71% of patients with beta-thalassemia [19]. With regards to pediatric patients, cardiac iron has been reported to be detected in one third of beta-thalassemia children aged 15–18 years, however, most remain asymptomatic [8].

As adherence to iron chelation therapy is a prerequisite for limiting iron overload complications and improving patient survival, efforts to optimize tolerability of chelation have been made [18]. The once-daily DFX DT has been an improvement in terms of convenience compared to parenteral deferoxamine and thrice-daily oral deferiprone, while retaining a safe and efficacious overall profile [17]. Numerous clinical trials comparing DFX DT to other iron chelators have demonstrated similar effects to deferoxamine with regards to efficacy, but with a compliance rate superior to deferoxamine and deferiprone [12]. As of recent, the new DFX FCT formulation has aimed for an even more favorable profile, offering better tolerability and palatability and, subsequently, potential for compliance.

A clinical trial comparing the two DFX formulations given over a 6-month period in 150 patients, both adults and children older than 10 years of age, has reported on the new formulation’s safety profile and pharmacokinetic properties, as well as patient related outcomes [20]. A longer, 2-year clinical trial provided additional data regarding long term DFX FCT efficacy and safety in children and adults. However, only three pediatric patients were enrolled, while drug efficacy was only assessed by serum ferritin [21]. Quarta et al. reported on real world data, evaluating 139 adult patients switching from DFX DT to FCT, demonstrating improved adherence to treatment and transfusional burden, without increased toxicity [17]. To our knowledge the current study is the first real-life report on DFX FCT safety and efficacy in pediatric thalassemia patients over 2 years of age, with consideration to all recommended clinical, laboratory and imaging monitoring parameters.

Given the reported higher bioavailability of DFX FCT compared to DT (as reflected by a 30% higher peak serum concentration), guidelines recommend an approximately 30% decrease in starting dose when switching to DFX FCT [9,13]. However, it is known that, drug absorption, distribution, metabolism and excretion generally present differences in the pediatric patients compared to adults [22]. A recent population pharmacokinetics study that included pediatric patients having undergone allogeneic hematopoietic stem cell transplantation demonstrated that extrapolation in terms of dosing might be inaccurate for pediatric patients [23]. In the present study, the median recommended starting DFX FCT dose had to be significantly increased during the course of study based on monthly follow ups in order to ensure desired outcomes (*p* = 0.011).

With regards to safety, DFX therapy has been associated to well characterized renal adverse events, which are generally mild, non-progressive and reversible [20]. Clinical trials have demonstrated mild increases in sCr in more than one third of patients (36.4–39.7%) [9]. Due to the known DFX renal toxicity, in case of a sCr value elevation of more than 33% compared to baseline in the course of treatment, a dose reduction is recommended [13]. In the present study, five (20%) patients presented with an increase in sCr of more than 33% compared to baseline at some point during the 12-month follow up. DFX FCT dose adjustments resulted in sCr normalization in four (16%) cases, whereas one (4%) patient had to discontinue DFX FCT due to persistently abnormal values. No correlation to patient age with sCr values was found, although patients presenting with sCr increase of more than 33% presented with higher body weight.

In the context of renal related side effects, pediatric reports have demonstrated proximal tubular dysfunction, i.e., low molecular-weight proteinuria and/or hypercalciuria [7]. DFX at therapeutic doses leads to exacerbation of hypercalciuria in a dose-dependent manner and requires monitoring for osteoporosis and urolithiasis, as well as other aspects of renal dysfunction [24]. Almost half of the patients in the present study presented with mild proteinuria during the 12-month follow up, which was reported as transient in all but one. Of note, at the end of study mean spot urinary protein: creatinine ratio showed no statistically significant change compared to baseline. On the other hand, while one third of patients presented with calcinuria at study entry, calcium: creatinine ratio increased beyond ULN in the vast majority (96%) during follow up, with 60% presenting a persistently abnormal value. In addition, while renal ultrasound was normal in all patients at study entry, including those presenting with calcinuria, 2 (8%) patients developed imaging signs of nephrolithiasis during follow up.

The literature has recognized low iron burden as a risk factor for tubular dysfunction. Excessive rapid iron removal modifying renal hemodynamics, as well as direct drug toxic effect with tubular necrosis, have been associated to that end [6]. The present study found no correlation between ferritin levels and proteinuria or calcinuria. Recently, DFX-induced mitochondrial swelling has been reported as an off-target, unrelated to iron metabolism effect, involving the proximal tubule which has very high density of mitochondria. This explains why other chelators do not produce similar toxicity. DFX binding to iron or albumin appeared to reduce toxicity in vitro, suggesting that patient iron load and nutritional status might play some role in determining baseline risk of toxicity [25].

With regards to hepatotoxicity, liver enzymes have been reported to be increased in a considerable number of pediatric patients receiving DFX DT, however, the majority already presented with elevated baseline alanine aminotransferase [7]. In the present study, baseline transaminase values were normal in all patients, almost half of them presenting with liver enzyme elevation at some point during follow up. In 3 (12%) patients persistently abnormal values led to DFX FCT discontinuation. Of importance, these patients represented the entire 2–6-year group. Other liver markers, such as bilirubin and ALP, showed no deviation from baseline values in the present study. A literature case report has recently described an adult thalassemic patient, previously well tolerating DFX DT, demonstrating hepatotoxicity and Fanconi-like syndrome when switching from DFX DT to FCT [26].

Furthermore, similar to deferoxamine, sensorineural hearing loss (SNHL) and hypoacusis have been reported in patients on DFX [13]. Ototoxicity occurs due to damage of the ciliated cells of the basal turn of the cochlea, causing high-frequency SNHL. For determining hearing loss secondary to drug ototoxicity, PTA and DPOAE testing show a reasonable level of reliability [27]. In the present study, evaluation with PTA demonstrated mild hearing loss in 3 (12%) patients prior DFX FCT administration, which resolved until study end, while another (4%) patient presented mild hearing loss during the 12-month DFX FCT treatment. Literature case reports have, also, demonstrated abnormal ocular findings, such as decreased vision, retinopathy and lens opacities attributed to DFX use [6]. In the present study, fundoscopy revealed diffuse retinal pigmentation in one (4%) patient.

The commonest adverse events described in DFX DT studies have been related to the gastrointestinal tract and are present in 15.2% to 45.6% of patients. Second in frequency, skin rash has been reported in 8.7% to 13.6% of patients receiving the drug [9]. Comparison between DFX DT and FCT formulations has shown lower frequency of severe gastrointestinal adverse events in patients receiving FCT (DT 12.8% versus FCT 4.6%) [20]. In the present study, only mild gastrointestinal events were reported in 16% of patients after DFX FCT initiation, resolving quickly without intervention, whereas skin rash occurred in only one (4%) patient. None of the above adverse events led to a need for FCT discontinuation.

In terms of chelation discontinuation due to adverse events, a meta-analysis associated DFX therapy to the lowest rate (0.2%). Events leading to discontinuation were rash, gastrointestinal symptoms and transaminase elevation [6]. In the present study, abnormal liver enzyme and sCr values led to DFX FCT discontinuation in 3 and 1 patient, respectively.

In order to keep a balance between iron load and chelator toxicity, maintaining serum ferritin levels between 500 and1000 ng/mL would be preferable, given that these levels are associated with complication free survival [7,28]. Serum ferritin measurement is inexpensive and, therefore, widely used for monitoring iron overload in thalassemic patients. However, the measurement has well known limitations, mainly related to absence of reliability in inflammatory, infectious, autoimmune and, even, stress conditions [18]. In the present study, ferritin levels remained relatively stable during patient follow up. Mean ferritin values were negatively correlated to patient age, weight and height at study entry but showed no correlation with mean DFX dose. Of interest, patients older than 10 years presented with significantly lower mean ferritin values (*p* = 0.023). This could, possibly, be attributed to the fact that ferritin, as an acute phase protein, is found elevated in infections, which are much more common in children of school or pre-school age. 

Different body organs are known to be affected in different ways by iron deposition, depending on underlying iron load mechanism and rate of accumulation. Beyond serum ferritin, non-invasive imaging techniques help quantify iron overload in various organs and monitor clinical response to chelation therapy [18]. MRI is highly sensitive and offers the possibility of frequent and direct assessment of iron in target organs, while results are considered equivalent to biopsy LIC [8,29]. Annually monitoring with MRI imaging in children is recommended by the age of 8–10 years on words, or as early as it is feasible without sedation [7,29]. DFX has been reported to have dose dependent effect on LIC and, even, myocardial T2*, allowing for patients to move to lower cardiac risk categories [7,18,30]. In the present study, MRI monitoring revealed a statistically significant increase in cardiac T2*, with simultaneous reduction in LIC values during the 12-month follow up. Similar to previous studies, DFX proved effective in protecting from organ iron overload.

## 5. Conclusions

In general, DFX FCT administration proved safe in older children, but younger patients experienced adverse events-especially in terms of increased liver enzyme values, which failed to respond to dose adjustments. However, one should bear in mind the small sample size when assessing these results. With regards to renal function, a sCr increase was observed in lower rates than previously reported [9]. Of note, evidence of tubular dysfunction, especially calcinuria, was reported in almost all patients, requiring close monitoring. With regards to efficacy, DFX FCT proved to be effective in maintaining a stable iron load in the transfusion dependent patients. As for DFX FCT dosing, higher doses than generally recommended for drug initiation were required in order to ensure adequate iron load balance-possibly, suggesting variability in bioavailability in children and adolescents.

## 6. Patents

The authors agree that the copyright of their article is transferred to the publishers. No patent application is related to the manuscript.

## Figures and Tables

**Table 1:** Patient demographics and chelation treatment at study entry.

	Study Entry (*n* = 25)
Male: Female, *n*	16:9
Median age (range), years	13 (2–18)
Age groups, *n* (%)	
2–6 years old	3 (12%)
6.1–10 years old	5 (20%)
10.1–18 years old	17 (68%)
Median height (range), centimeter	148.5 (95–183)
Median weight (range), kilograms	39 (13.6–70)
Previous chelation, *n* (%)	
Yes	21 (84%)
No	4 (16%)
DFX prior to study, *n* (%)	
Yes	14 (56%)
No	7 (28%)

**Table 2:** Laboratory values prior to and 12-months after DFX FCT treatment.

Variable	Study Entry	Study End	
Median DFX FCT dose (range), mg/kg/d	14.3 (10–22.5)	18 (7.5–22.5)	*p* = 0.011
Median serum ferritin (range), ng/mL	1143 (345–2253)	1117 (318–3256)	*p* = 0.510
Median sCr(range), mg/dL	0.65 (0.4–0.9)	0.66 (0.45–0.99)	*p* = 0.165
Median GFR (range), mL/min	93 (72–141)	92 (71–139)	*p* = 0.188
Spot urinary protein:creatinine ratio	0.06	0.06	*p* = 0.547
Spot urinary calcium:creatinine ratio	0.16	0.21	*p* = 0.100
AST (range), U/L	22.5(12–49)	22 (14–111)	*p* = 0.173
ALT (range), U/L	14 (6–31)	15 U/L (7–127)	*p* = 0.076
Bilirubin (range), mg/dL	1.95 (0.61–10.8)	1.79 (0.91–14.81)	*p* = 0.089
ALP (range), U/L	137 (71–506)	159 (59–445)	*p* = 0.710
LIC (range), mg/g/dry weight	4.86 (1.1–12)	4.01 (1.4–13.75)	*p* = 0.142
Cardiac MRI T2*, ms	28.8 (21.5–38.3)	33.5 (22.3–68.2)	*p* = 0.006

DFX FCT: deferasirox film coated tablet, sCr: serum creatinine, GFR: glomerular filtration rate, AST: aspartate aminotransferase, ALT: alanine aminotransferase, ALP: alkaline phosphatase, MRI: magnetic resonance imaging.

## Data Availability

The data presented in this study are available on request from the corresponding author.
